# Parvalbumin Gene: A Valuable Marker for Pike Authentication and Allergen Risk Assessment

**DOI:** 10.1021/acs.jafc.4c01410

**Published:** 2024-05-23

**Authors:** Eliška Čermáková, Subham Mukherjee, Denisa Nováková, Petra Horká, Kamila Zdeňková, Kateřina Demnerová

**Affiliations:** † Department of Chemistry, Biochemistry and Food Microbiology, Food Research Institute Prague, Radiová 1285/7, Prague 10 102 00, Czech Republic; ‡ Department of Biochemistry and Microbiology, University of Chemistry and Technology, Prague, Technická 5, Prague 6 166 28, Czech Republic; § Lennard-Jones School of Chemical and Physical Sciences, Keele University, Staffordshire ST5 5BG, United Kingdom; ∥ Institute for Environmental Studies, Faculty of Science, Charles University, Benatska 2, Prague 2 128 01, Czech Republic

**Keywords:** food allergy, DNA, *Esox*, food fraud, PCR, LAMP

## Abstract

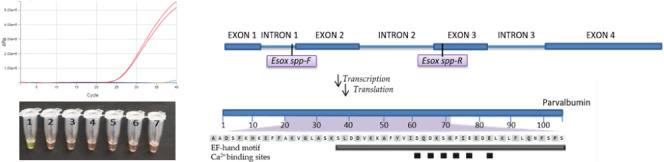

Fish from the pike (*Esox*) genus are valued in gastronomy for their superior meat quality. However, they can cause allergic reactions in sensitive consumers. This work aimed to fill the gap in the detection of pike allergens using molecular–biological techniques. New, fast, and accurate loop-mediated isothermal amplification (LAMP) and real-time PCR (qPCR) assays were designed to detect pike DNA using the parvalbumin gene as a marker. LAMP was assessed by electrophoresis, SYBR green optical detection, and real-time fluorescence detection. The latter was the most sensitive, detecting as little as 0.78 ng of pike DNA; the qPCR detection limit was 0.1 ng. The LAMP analysis took 20–70 min, which is significantly faster than qPCR. The study provides reliable detection and quantification of the parvalbumin gene in both fresh and processed samples and further highlights the versatility of the use of the parvalbumin gene for the authentication of food products and consumer protection via refined allergen risk assessment that is independent of the type of tissue or food processing method used.

## 1 Introduction

Fish from the pike family (*Esocidae*) are an important freshwater predators with a Holarctic distribution.^1^ The most significant genus within this family is genus *Esox*, which includes seven species of pike: American pickerel (*Esox americanus* Gmelin, 1789),^2^ Amur pike (*Esox reichertii* Dybowski, 1758*)*,^3^ Aquitanian pike (*Esox aquitanicus* Denys, Dettai, Persat, Hautecoeur and Keith, 2014),^4^ chain pickerel (*Esox niger* Lesueur, 1818),^5^ muskellunge pike (*Esox masquinongy* Mitchill, 1824),^6^ northern pike (*Esox lucius* Linnaeus, 1758), and southern pike (*Esox cisalpinus*/*E. flaviae* Bianco and Delmastro, 2011). The southern pike was considered a special color mutation of *E. lucius* until 2011 when it was declared a separate species based on DNA analyses.^7,8^ The most widespread pike species is *E. lucius*, which is found in the freshwater and brackish coastal waters of four continents: Asia, Europe, North America (native occurrence), and Africa (introduced occurrence).^9,10^ In nature, *E. lucius* interbreeds with other pike species, such as *E. niger*(5) or *E. masquinongy*, to form hybrids like a tiger muskellunge.^11,12^ Genetically, the individual pike species are, thus, very similar.

Pike species have analogous ecological behaviors as a sit-and-wait ambush predator, important for freshwater ecosystems. They are valued for their superior meat quality and suitability for aquaculture. They are also sought after by fishermen and serve as an excellent source of food.^4,13,14^ In the Czech Republic, for example, northern pike ranks among the top consumed fish due to its delicate taste and gastronomic appeal, despite its seasonal availability and higher market price compared with other common species like trout or carp. However, pike is usually sold in the form of fillets. The processed nature of its meat makes it prone to adulteration, such as undeclared substitution of fish species, posing health risks, especially for those with fish allergies. Adulteration of fish meat, estimated to be as high as one-third of traded commodities in the European Union,^15^ underscores the importance of reliable species identification in any sample of fish meat, even heat treated or without morphological features, crucial for consumer health and economic integrity.

It is known that allergic reactions to fish in humans are caused by proteins that contain at least two or more IgE binding sites.^16,17^ By January 2024, 13 such proteins had been registered in the WHO/IUIS allergen nomenclature subcommittee database (http://www.allergen.org), including parvalbumin, collagen, tropomyosin, or aldolase. β-parvalbumin, an acidic sarcoplasmic calcium-binding protein, is considered the main fish allergen.^17^ However, to the best of our knowledge, no article has yet been published that deals with the detection of allergens in pike species, and little is still known about pike parvalbumin.

From another point of view, the development of methodologies for the detection of pike species, especially northern pike, is important because it is a significant invasive species,^18^ whose presence can affect the management of other fish species.^19^ Mitochondrial genes, such as cytochrome *b* or cytochrome oxidase 1, are typically used to investigate the evolution, phylogeography, and genetic diversity of the population. Due to this, the mitochondrial genomes of pike are well-known.^3,20−26^ In contrast to that, an annotated whole genome sequence (anchored on chromosomes) is still available only for *E. lucius*.^27^ Nuclear gene sequences are therefore not well-known in a pike, and the development of methodologies for their detection may be more difficult.

For successful control of food fraud in the market, the early identification of fish species is essential. A possible solution for fast detection could be the polymerase chain reaction (PCR) or loop-mediated isothermal amplification (LAMP) method, which is generally presented as a robust, sensitive, and easy method without demanding laboratory equipment.^28,29^ LAMP was developed by Notomi et al.^30^ in 2000 and has since been commonly used for the detection of GMOs in food and feed,^31^ as well as for the identification of pathogenic microorganisms.^32,33^ The use of LAMP for the detection and identification of allergenic foods has also been published.^34−36^ However, to date, LAMP has been used very little to identify fish species, and to our knowledge, no study has addressed the detection of fish allergens.

The present work selected northern pike (*E. lucius*), a type species of the genus *Esox*, as a case study for the development of a new LAMP and real-time PCR assays and their comparison. Since parvalbumin is a major fish allergen but little is known about it in pike, we used the nuclear gene encoding this allergen, parvalbumin, as a genetic marker. To the best of our knowledge, this is the first report on LAMP and PCR assays for the detection of pike DNA for allergen prevention and food quality control.

## 2 Materials and Methods

### 2.1 Sample Collection

We examined 35 animal species belonging to the 12 families of ray-finned fishes and 7 families of birds, bivalves, and mammals. All tested tissue samples are listed in Table 1; list of analyzed commercial samples is given in Table 2.

**Table 1:** List of Analyzed Samples

scientific name	common name	type of specimen
Fish Species		
*Anarhichas lupus*	Atlantic wolf-fish	frozen fillet
*Coryphaena hippurus*	mahi-mahi (purple cod)	frozen fillet
*Cyprinus carpio*	common carp	whole fish
*Esox aquitanicus*	aquitanian pike	muscle tissue preserved with ethanol
*Esox cisalpinus*	southern pike	clipped fin, muscle tissue preserved with ethanol
*Esox lucius*	northern pike	whole fish, frozen fillet
*Gadus morhua*	Atlantic cod	frozen fillet
*Hippoglossus hippoglossus*	Atlantic halibut	frozen fillet
*Lophius budegassa*	black-bellied angler	whole fish
*Lophius piscatorius*	anglerfish	whole fish
*Merluccius hubbsi*	Argentine hake	frozen fillet
*Merluccius productus*	Pacific hake	frozen fillet
*Oncorhynchus keta*	chum salmon	frozen fillet
*Oncorhynchus mykiss*	rainbow trout	whole fish
*Oncorhynchus nerka*	sockey salmon	frozen fillet
*Oreochromis niloticus*	Nile tilapia	frozen fillet
*Pollachius virens*	saithe	frozen fillet
*Salmo salar*	Atlantic salmon	frozen fillet
*Salmo trutta*	brown trout	frozen fillet
*Salvelinus alpinus*	Alpine char	whole fish
*Sander lucioperca*	European pikeperch	whole fish
*Scomber scombrus*	Atlantic mackerel	whole fish
*Solea solea*	black sole	frozen fillet
*Thunnus albacares*	yellowfin tuna	frozen fillet
*Thunnus thynnus*	Atlantic bluefin tuna	frozen fillet
Nonfish Species		
*Anas platyrhynchos* f. domestica	domestic duck	muscle tissue
*Bos primigenius taurus*	domestic cattle	muscle tissue
*Cervus elaphus*	red deer	muscle tissue
*Dama dama*	European fallow deer	muscle tissue
*Gallus gallus domestica*	domestic chicken	muscle tissue
*Macrobrachium rosenbergii*	giant river prawn	whole frozen crustacean
*Meleagris gallopavo* f. domestica	domestic turkey	muscle tissue
*Mytilus edulis*	blue mussel	muscle tissue
*Sus scrofa*	wild boar	muscle tissue
*Sus scrofa domesticus*	domestic pig	muscle tissue

**Table 2:** List of Analyzed Commercial Samples

name	declared species of fish	content	place of catch
skinless pike fillet	northern pike (*Esox lucius*)	-	Russia
Lemberg pike caviar		pike roe (*Esox lucius*), salt, E415	not specified
dried pike fish		pike and salt	Kazakhstan
dried, gutted pike fish		pike and salt	Pacific Ocean

### 2.2 Sample Preparation and DNA Extraction

The tissue samples were homogenized using an analytical grinder; 200 mg was then weighed out for subsequent isolation. From pike samples (muscle, part of a fin) preserved in 96% ethanol, a piece of tissue or fin was cut off and weighed similarly to the other samples. Then, 50 mg of glass beads (BioSpec Products Inc., USA) with a size of 0.6–0.8 mm and 650 μL of CTAB extraction buffer (20 g·L^–1^ CTAB, 1.4 M NaCl, 100 mM Tris, 20 mM EDTA; pH adjusted to 8.0) preheated to 65 °C were added to all samples. The mixture was thoroughly mixed using a FastPrep (MP Biomedicals, USA) and then incubated with constant stirring for 30 min at 65 °C to disintegrate the cells. Subsequently, 10 μL of proteinase K solution (20 mg·m^L–1^) was added to the suspension, and it was incubated for the next 30 min at 65 °C followed by centrifugation for 10 min at 12000*g*. The supernatant was transferred to a new microtube containing 650 μL of chloroform; the mixture was shaken and then centrifuged for 15 min at 12000*g*. The entire volume of the DNA-containing solution was transferred to a new microtube, and one 260 μL of CTAB precipitation buffer (5 g·L^–1^ CTAB, 40 mM NaCl) was added. The solution was mixed by pipetting and incubated for 60 min at room temperature without stirring. After incubation, the solution was centrifuged (15 min, 12000*g*), the supernatant was decanted, and the precipitate was dissolved by adding 350 μL of 1.2 mol·L^–1^ NaCl solution. Subsequently, an equal volume of chloroform was added to the solution; the mixture was shaken and centrifuged (15 min, 12000*g*). The aqueous phase, containing nucleic acids, was transferred to a new microtube comprising 180 μL of isopropanol. The solution was mixed by inverting the microtube and incubated for 20 min at room temperature. After centrifugation (15 min, 12000*g*), the supernatant was decanted, and 500 μL of ethanol (70% solution) was added to the resulting DNA pellet. The microtube was mixed by inverting it and centrifuged (10 min, 12000*g*). The supernatant was then discarded, and the pellet was dried at 37 °C. The dried DNA pellet was subsequently resuspended in 50 μL of nuclease-free water.

The quality of the isolated DNA was verified spectrophotometrically (NanoDrop One, Thermo Scientific) and electrophoretically on a 1% agarose gel.

### 2.3 *In Silico* Analysis of the *Esox* Species Parvalbumin Gene

The genome sequences of *Esox* species available in the National Centre for Biotechnology Information (NCBI) databases (https://www.ncbi.nlm.nih.gov/) were analyzed to search for parvalbumin gene sequences. Genome analysis was performed in Geneious Prime software (V2021.1.1) with medium sensitivity settings. The genome sequences of *Danio rerio* (GCA_000002035.4) and *Esox lucius* (GCA_011004845.1) were used as a reference parvalbumin gene set comprising 16 parvalbumin genes in total. The genomes downloaded from *Esox niger* (GCA_016801105.1) and *Esox masquinongy* (GCA_016801175.1) were mapped to the reference data set and manually screened for the number and position of the parvalbumin genes. Then, the parvalbumin gene sequences were extracted for both genomes and aligned using the MAFFT alignment (MAFFT v7.450)^37,38^ for subsequent analysis. The analysis of selected genes was also done simultaneously using Ensembl Rapid Release tools (https://rapid.ensembl.org/); proteins were also analyzed in UniProt (https://www.uniprot.org/).

### 2.4 Primer and Probe Design

**Figure 1:**
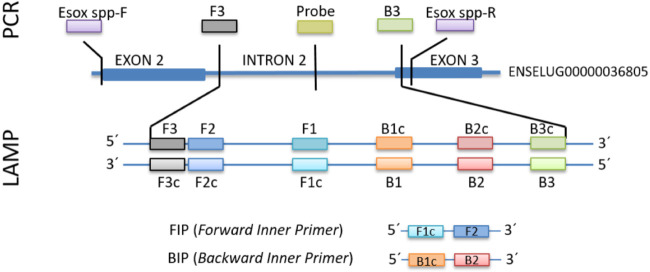
Schematic diagram of PCR (upper part) and LAMP (lower part) primers placement in the *Esox lucius* genome (specifically ENSELUG00000036805 gene).

LAMP primers (outer: F3, B3; inner: FIP, BIP) specific to the pike parvalbumin gene were designed by using the EIKEN Primer Explorer web tool (http://primerexplorer.jp). Loop primers were not used in the analysis. On the contrary, outer primers (EsoxLu-F3 and EsoxLu-B3) were also used in the qPCR assay. For higher specificity, an arrangement with a TaqMan hydrolysis probe was used, in which selected bases were modified by lock nucleic acid (LNA) treatment due to an increase in the melting temperature. The locations of the primers in the pike genome are schematically shown in Figure 1.

To verify the amplifiability of fish DNA, primers from the study by Sun et al.^39^ annealing to the fish parvalbumin gene were used. Primers amplifying the gene for myostatin were chosen to check the amplifiability of mammalian and bird DNA.^40^

All oligonucleotides (listed in Table 3) were synthesized by EastPort Praha (Czech Republic) in MassCheck quality.

**Table 3:** Overview of Used Primers

primer name	primer sequence (5′–3′)	product length	Ta^c^	reference
Esox spp-F	TCTCCCTACAGCTGCTGACT	412 bp^b^	60 °C	This study
Esox spp-R	TGGGGAGAAGTTCTGCAGGA			
EsoxLu-F3	GGAATCTAACTCCTACTATTGC	F3–B3 primers provide 221 bp^b^ length product	63 °C for LAMP; 60 °C for PCR	This study
EsoxLu-B3	AACAGCCTGGATGGGTAC			
EsoxLu-F2	GGTTTGACTTGCTCCCTC			
EsoxLu-F1c	AGTGCTTTAAGTTCACTCTAAGCAT			
EsoxLu-B2	AAACTATGCACCAGTTTTCCT			
EsoxLu-B1c	AATGTTGGGGCAAAAAAGAGCA			
Esox_P2^a^	FAM-ATG[+T]TG[+G]GGCAA[+A]AAAGAGCAGAACTTTAA-BHQ1			
Sun_F	CAGGACAAGAGTGGCTTCAT	app. 300 bp	55 °C	Sun et al.^39^
Sun_R	GAAGTTCTGCAGGAACAGCTT			
My_F	TTGTGCAAATCCTGAGACTCAT	97 bp	60 °C	Laube et al.^40^
My_R	ATACCAGTGCCTGGGTTCAT			

a LNA modification of bases is marked with a sign plus (+).

b Product length was determined for E. lucius (GCA_011004845.1).

c Ta is the annealing temperature of the primer pair.

### 2.5 Real-Time PCR Assay

In this work, two approaches to the detection of PCR products were verified, namely, the use of an intercalation fluorescent dye and a TaqMan hydrolysis probe.

In the first case, 5×x HOT FIREPol EvaGreen qPCR Supermix (Solis Biodyne) was used. The concentration of each primer was 200 nM in the reaction; the amount of DNA added to the reaction was 100 ng. The total reaction volume was 20 μL. The temperature program was set according to the parameters given by the manufacturer and the suitable primer annealing temperature (Ta) given in Table 3: initial denaturation for 12 min at 95 °C, then 40 cycles of 95 °C for 15 s, Ta °C for 20 s, 72 °C for 30 s, and subsequent polymerization for 5 min at 72 °C. The melting curve was then measured.

GoTaq Probe qPCR Master Mix was used for the qPCR assay with the probe. The composition of the reaction mixture and the temperature cycle were according to the manufacturer’s recommendations. The concentration of oligonucleotides was 400 nM each primer and 250 nM LNA probe in the reaction. 100 ng of DNA was added to the reaction. The total reaction volume was 20 μL. The temperature program included an initial denaturation at 95 °C for 2 min, followed by 40 cycles of 95 °C for 15s and Ta °C for 60s.

In both cases, PCRs were performed on a QuantStudio 5 and StepOne plus thermocycler; the measured data were evaluated by using the Design and Analysis 2.6.0 software.

### 2.6 LAMP Assay

LAMP reactions were performed in a 25 μL volume consisting of 50 ng of DNA template, 320 U/mL *Bst* 3.0 DNA polymerase (New England Biolabs, Ipswitch, MA), 4 mM MgSO_4_, 1.4 mM mixture of dNTP, 0.4 μM both inner primers (FIP, BIP), and 0.08 μM of each outer primer (F3, B3).

Amplification was carried out in a Biometra T-Gradient thermocycler at 63 °C for 60 min. Reactions were terminated by a subsequent enzyme inactivation at 80 °C for 2 min. After that, the amplification products were detected electrophoretically on 2% agarose gel and/or using a SYBR Green I (10000×) dye when 0.2 μL of concentrated dye was added to each tube, and a color change was observed under daylight and fluorescence under UV radiation.

For real-time LAMP, the same reaction mixture as in the previous case was mixed, only with the addition of 0.5 μL of LAMP Fluorescent Dye (New England Biolabs) instead of nuclease-free water. Amplification was performed at 63 °C for 60 min (set as 60 cycles of 1 min) and finalized at 80 °C for 2 min. The increase of fluorescence was monitored on the SYBR channel in a real-time thermocycler ABI 7500 (Applied-Biosystems), with fluorescence detection after each cycle.

### 2.7 Specificity and Sensitivity of Analysis

The specificity of the designed oligomers was tested on a wider panel of organisms (Table 1). In the case of real-time LAMP, only the amplifiability of pike DNA was verified; DNA from selected fish species and nuclease-free water were used as a negative control.

Using the selected combination of oligonucleotides, we further estimated the limit of detection (LOD) for both the LAMP and qPCR protocols. An estimate of the limit of detection was obtained through a dilution series (4×) of pike DNA; the starting concentration of the isolate was 50 ng·μL^–1^. Furthermore, a series of mixtures of pike (*Esox lucius*) and cod (*Gadus morhua*) tissues were also prepared for the estimation of the detection limit. The mixture was prepared with a total weight of 10 g; the representation of one species was in the range of 5–95 wt %.

## 3 Results and Discussion

### 3.1 Results of *In Silico* Analysis of *Esox* Parvalbumin Genes

**Figure 2:**
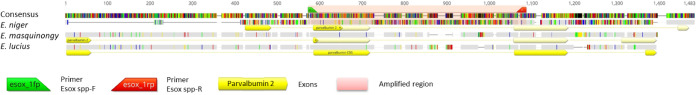
Alignment of parvalbumin β2 genes in three pike species: *Esox niger* (JACXGJ010000162.1), *Esox masquinongy* (JACXGI010000598.1), *Esox lucius* (CM002830). The nucleotide sequence is shown in color in Consensus, where green = T, blue = C, red = A, and yellow = G. For partial sequences, only the SNPs are colored. The *Esox* spp-F (green) and the *Esox* spp-R (red) primers used for Sanger sequencing are also aligned. The dark yellow color of exons denotes sequences present in the NCBI database, highlighting their verified status, while light yellow signify sequences not currently available or identified in the database as part of the parvalbumin gene.

Analysis of *E. lucius*, *E. niger*, and *E. masquinongy* genomes showed a low diversity of parvalbumin among them. *In silico* analysis revealed that *E. lucius* and *E. niger* have 7 parvalbumin genes, consisting of 2 parvalbumin α and β1, and 3 parvalbumin β2, while the genome of *E.masquinongy* comprises only 6 parvalbumin genes (2 gene copies of parvalbumin α, β1, β2). Since parvalbumin β2 is the main cause of allergy in humans, we have focused on it in our work. First, the nucleotide sequences of the parvalbumin β2 gene obtained from the available pike genomes were compared. The parvalbumin β2 gene exhibited more than 85% similarity among these three species, as illustrated in Figure 2.

**Figure 3:**
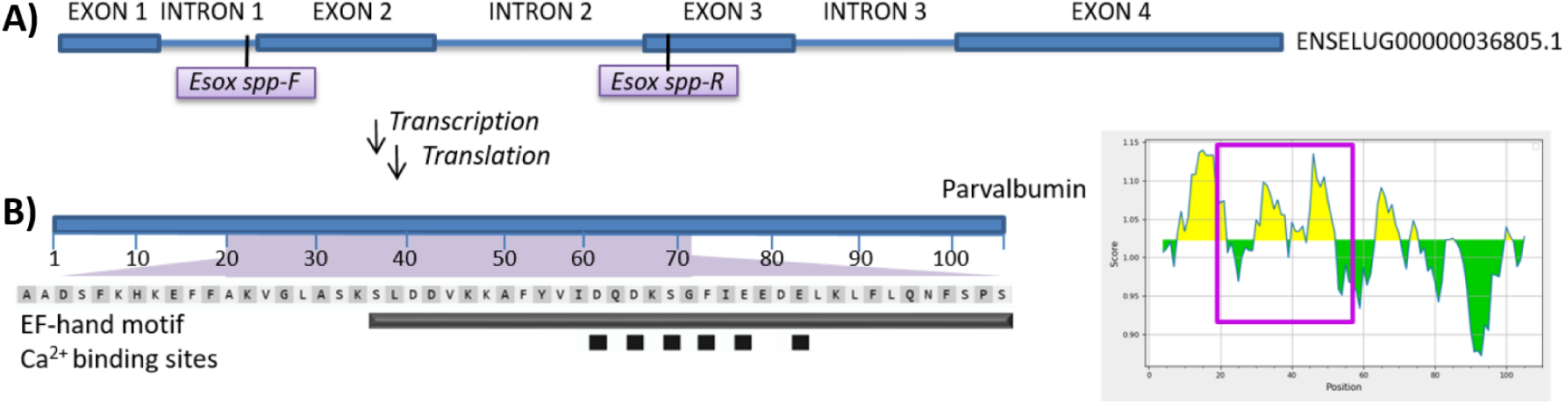
Schematic of the analyzed part of the parvalbumin gene (*pvalb*) and its overhang to protein analysis. (A) Schematic illustration of the *pvalb* gene, namely ENSELUG00000036805.1; (B) results of the parvalbumin protein *in silico* analysis: position of the EF-hand motif and the binding sites (left) in the amplified area; analysis of parvalbumin antigenicity (right) according to Kolaskar and Tongaonkar.^43^*In silico* analysis results are based on data available in the ENSEMBL, NCBI and UniProt databases. The purple box encloses the region defined by the *Esoxspp* primers.

Furthermore, a pair of universal primers for pike DNA was designed; the length of the amplicon was approximately 412 bp (425 bp for *E. niger*). As the second intron of the parvalbumin gene appears to be a promising platform for fish species identification,^41,42^ targeted region ranged from the 3′ end of the first intron to the 5′ end of the third exon of the parvalbumin gene (Figure 2). This approach allows the amplification of a large part of the nucleotide sequence encoding the EF-hand motif in the parvalbumin protein, including binding sites for calcium ions in *E. lucius* (Figure 3). Also, the average residue score of the amplified region is 1.02 according to the Kolaskar and Tongaonkar methods (http://tools.iedb.org) that predict the antigenic determinants on proteins.^43^

Furthermore, universal primers were used for sequencing part of the *E. cisalpinus* and *E. aquitanicus* parvalbumin genes. Based on the analysis of available sequencing data, we designed primers for LAMP and qPCR detection of European *Esox* species targeting the second intron of the parvalbumin β gene.

The NCBI Genome data viewer also found the possible position of *Esox* spp primers in the LOC105005800 gene in the *E. lucius* genome (chromosome 5). The expected product length is app. 450 bp. The similarity of this sequence and the amplified area of the ENSELUG00000036805.1 gene is 100%. Also, many orthologues have been *in silico* detected among fish parvalbumins (www.orthodb.org). However, the LOC105005800 gene is described as a probable calcium-binding protein and contains 9 exons, which is atypical for the parvalbumin gene.

Our experimental data showed that only one product is formed under the given reaction conditions (Figure S1). The Sanger sequencing confirmed a length of 411 bp, indicating the amplicon of the parvalbumin gene.

### 3.2 DNA Extraction and Quality Verification

The DNA from the species tested and the commercial products (Tables 1 and 2) was successfully extracted using the CTAB protocol, as described above. All DNA extracts were measured spectrophotometrically using a NanoDrop (Thermo Fischer, München, Germany) to determine DNA purity and concentration. The absorbance ratio A260/280 in all DNAs from the tissue samples was between 1.7 and 1.9, indicating good quality DNA. In the case of extracts from commercial products, the absorbance ratio was in the range of 1.8–2.0, which may indicate a higher presence of RNA. In a study by Piskata et al.,^44^ where several extraction methods for DNA isolation from food products with different technological processing were compared, it was stated that only the PCR method can definitively determine the quality of the isolated DNA. Similar results were published by Hellberg et al.^45^ and Čermáková et al.^46^ who verified the quality of the DNA obtained and its amplifiability by PCR.

Therefore, we used universal primers for fish,^39^ and mammalian and bird DNA^40^ amplification in this work. All of the DNAs were successfully amplified.

### 3.3 Evaluation of Primer Specificity

The specificity of the designed primers was evaluated as described above. First, the EsoxLu-F3 and EsoxLu-B3 primers (outer primers for LAMP) were verified by the qPCR method to see if they bind to the template DNA. After that, their combination with inner primers was tested by the LAMP.

**Figure 4:**
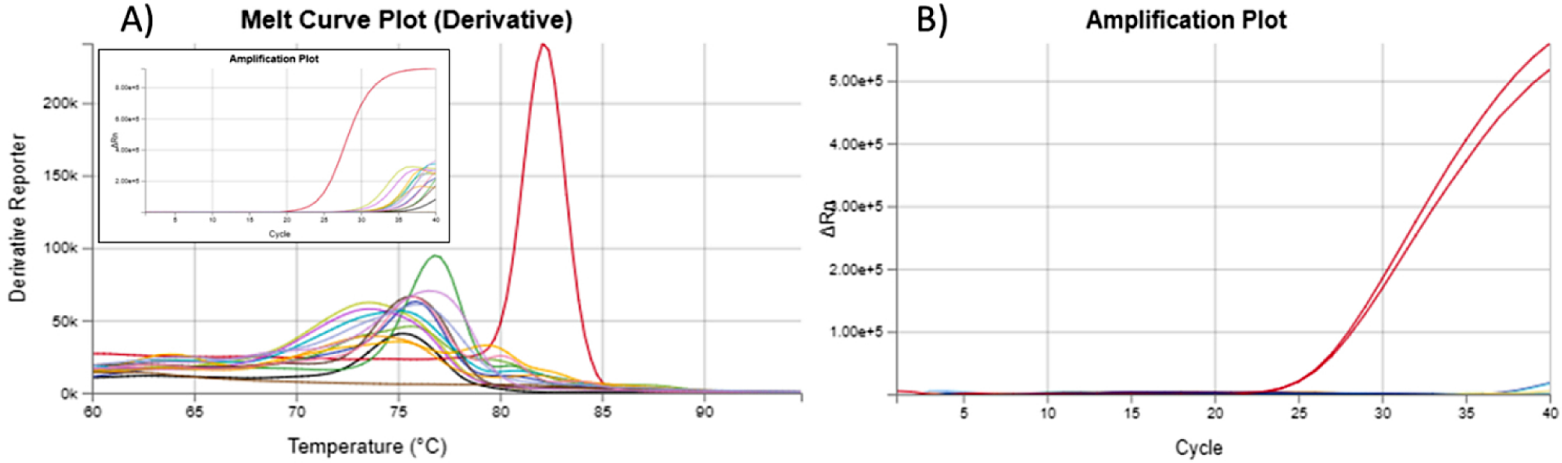
Evaluation of the qPCR assay specificity using Eva Green (A) and TaqMan probe (B) detection (*n* = 2, *N* = 5). Amplicons of northern pike DNA are represented by a red line; nontarget species are shown in other colors.

Primers F3–B3 were found to amplify the DNA of several nontarget fish species in qPCR assay (Ct ≥ 30) when mastermix with intercalating EvaGreen dye was used. Although pike DNA is amplified with a much higher fluorescence intensity in the amplification curves and a significantly lower Ct compared to other fish species, we do not consider the identification of the target species by Ct values to be reliable. However, the melting curve analysis of amplicons provided a unique peak to the pike genus; the melting temperature of the pike amplicon is 82.8 ± 0.4 °C. In addition, it is evident from the melting curve that the primers give only one product (Figure 4). Thus, nonspecific products are not formed, for example, due to the amplification of a different type of parvalbumin gene, as was expected based on the *in silico* analysis. This may allow not only reliable detection but also quantification of the parvalbumin gene in the test sample. Even so, a TaqMan probe was further designed to provide pike DNA-specific amplification and reduce the analysis time as well as to improve the conditions for possible quantification of the analyzed gene. It was necessary to increase the melting temperature of a designed probe using an LNA modification of the bases. LNA modification has also been shown to increase the sensitivity and specificity of oligonucleotides.^47^ Therefore, bases for modification were selected based on a comparison of amplicon sequences in amplified fish species. In addition to the aforementioned Esox_P2, we tested two other LNA probes (data not shown): Esox_P with less modification closer to the 3′ end of the sequence (ATGTTGGGGCAAAAAAG[+A]GCAGAA[+C]TTTAA) and shorter probe, Esox_P3, with even modification (A[+G]A[+G]C[+A][+G]AA[+C][+T]T[+T]AA). Finally, we chose the Esox_P2 that has the LNA bases placed more at the 5′ end of the oligonucleotide. It is in accordance with Levin et al.,^48^ who demonstrated that modifications at this position give the same or better results than conventional oligonucleotides, while LNAs at the 3′ end or evenly spaced increased the Ct values compared to the unmodified sequence. Moreover, the technical advantages like intense fluorescence for pike samples and higher annealing temperature accompanied by higher specificity were seen in this work.

Analysis of *E. lucius* originating from different European countries verified that there are no significant mutations at the primer and probe positions that would prevent primer annealing and amplification. Lucentini et al.^7^ investigated the differences between two groups of pike in Europe. Given the existence of phenotypic, genotypic, and geographical differences, their results supported the existence of two lineages, namely, the northern pike (*E. lucius*) and southern pike (*Esox flaviae*, also called *E. cisalpinus*). Therefore, we also tested *E. cisalpinus* samples from Italy; amplification also occurred, indicating the low within-species variability in the intron sequence of the parvalbumin gene to which primers were designed.

In addition, amplification products from qPCR were sequenced, and the regions of similarity were searched in NCBI and ENSEMBL databases to confirm the possibility of *Esox* spp. identification. It turned out that the databases match only the *E. lucius* genome, even though the sequences themselves show a high similarity to the *E. masquinongy* genome as well. This confirms the high similarity of *E. lucius*, *E. cisalpinus*, and *E. aquitanicus* and, also, the need to expand the data related to pike genomes in the databases.

**Figure 5:**
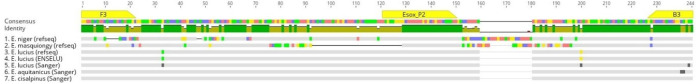
Alignment of qPCR amplicons (F3–B3 primers) with highly similar sequences searched in available pikes genomes. Color of nucleotides: green = T, blue = C, red = A, yellow = G.

Then, we manually searched for highly similar sequences and compared them with each other. The results confirmed the possibility of *Esox* species identification based on SNP and/or indels (see Figure 5), which could be useful for monitoring pike occurrence, as well as adulteration in the food industry. We also expect that the F3–B3 primers will not amplify the *E. niger*. This makes it possible to distinguish the European pike species (*E. cisalpinus*, *E. aquitanicus)* and *E. lucius* (Holarctic realm) from the American ones (*E. niger*, *E. masquiongy*). The nucleotide sequence similarity of pike amplicons is shown in Table S1. Unfortunately, the genome of *E. americanus* has not been published yet, and we have not been able to obtain a sample or DNA of this pike species. Thus, this is only a hypothesis based on significant differences between *E. niger*, *E.masquiongy* (available whole genome sequences), and pike species caught in Europe (amplicon sequences).

**Figure 6:**
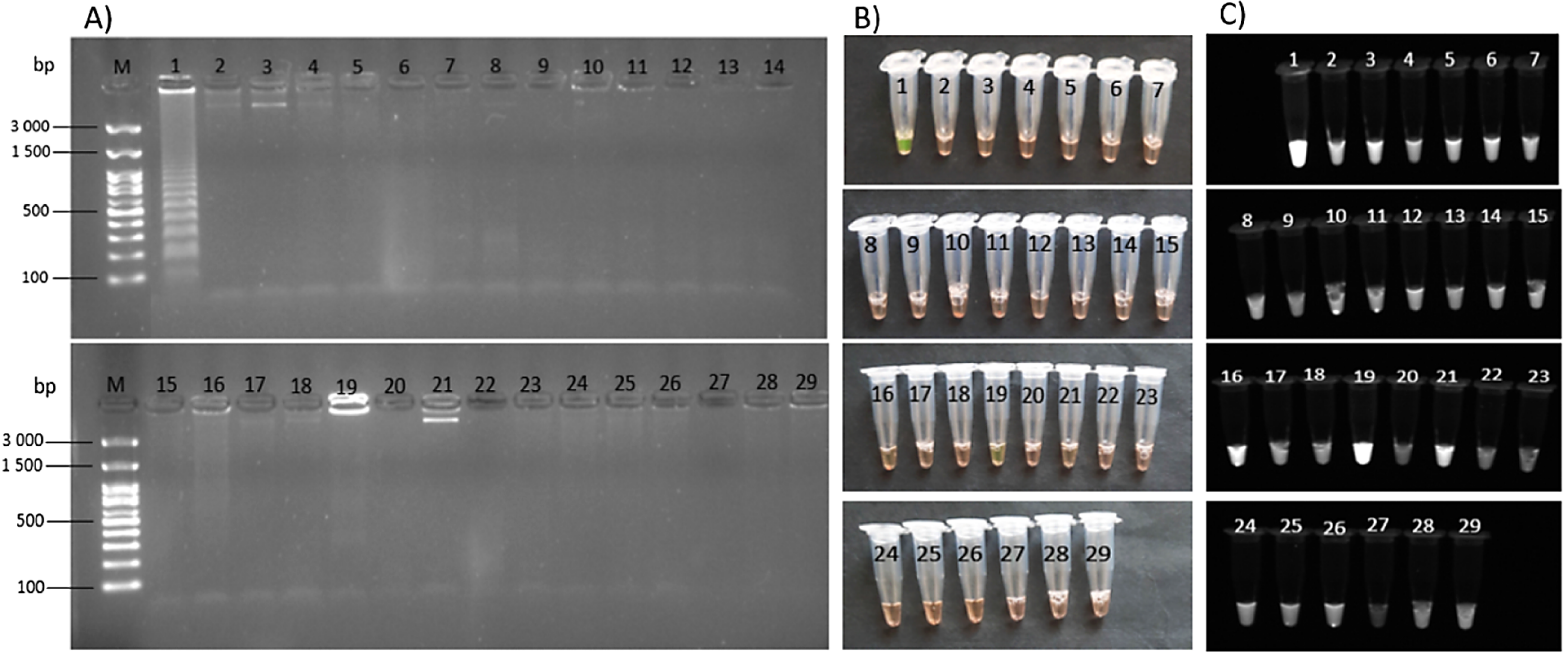
Visualization of LAMP products of *E. lucius* and nontarget species using gel electrophoresis (A) and SYBR Green dye under sunlight (B) and ultraviolet (C) light. M: 100 bp marker, 1: northern pike, 2: brown trout, 3: rainbow trout, 4: Atlantic salmon, 5: chum salmon, 6: sockey salmon, 7: Atlantic cod, 8: saithe, 9: anglerfish, 10: Argentine hake, 11: Pacific hake, 12: Atlantic mackerel, 13: Atlantic halibut, 14: European pikeperch, 15: Nile tilapia, 16: common carp, 17: Atlantic bluefin tuna, 18: yellowfin tuna, 19: Giant river prawn, 20: blue mussels, 21: domestic pig, 22: wild boar, 23: domestic cattle, 24: European fallow deer, 25: Red deer, 26: domestic turkey, 27: no template control, 28: domestic chicken, 29: domestic duck.

Subsequently, the specificity of the primers for LAMP was verified. The reaction products (lamplicons) were first detected by agarose gel electrophoresis. It can be concluded that the designed primers show high specificity for pike among the tested species since only the pike sample produces a characteristic band of products (Figure 6A). Also, after the addition of the intercalation dye SYBR Green I, the color changed from orange to green only in the microtube with the pike DNA. However, after the tubes were irradiated with UV light, more pronounced radiation also occurred in the DNA samples of giant river prawns (Rosenberg shrimp), common carp and domestic pig. These samples could then be evaluated as false positives, although, according to previous experimental results and *in silico* analysis of data available at NCBI, primer annealing should not occur in these cases. It is also evident from the electrophorogram that no lamplicons were formed in any of these samples. For this reason, detection under UV light for the proposed methodology cannot be recommended as the only evaluation method, and it should always be combined with at least an ophthalmic evaluation of the color of the reaction mixture under visible light (Figure 6).

It has also been observed that DNA samples isolated from *E. cisalpinus* fins show a change in the profile of the lamplicon “ladder” pattern. This could be due, for example, to a sequence change in the amplified DNA region. After analysis of the results, it can be assessed that this method shows high specificity for all pike representatives tested.

Finally, we tested the possibility of real-time detection of pike DNA samples by using the LAMP method. An increase in fluorescence was observed in all of the pike samples. However, for better detection specificity, we recommend designing the probes similarly to the qPCR.

Both PCR and LAMP amplified all of the pike species tested. Thus, it was confirmed that the selected section of the parvalbumin gene is sufficiently distinct to distinguish pike from other species and, at the same time, conservative enough to enable the detection of all tested pike with very good sensitivity (see below).

### 3.4 Comparison of the PCR and LAMP Results

While the PCR method has been used for fish identification for a long time, the LAMP method is still used more for the detection of fish parasites and other microorganisms.^49−52^ To the best of our knowledge, the use of LAMP for the identification of the fish itself has been published only for the members of salmon,^53,54^ trout,^55^ tuna,^29,56^ eel,^57^ cod,^58^ common sole,^59^*Arothorn* or *Diodon* genus.^60,61^ Thus, we believe that the present paper is the first to propose a LAMP protocol for pike species identification and, moreover, one of the few that use a nuclear gene as a marker since most published protocols have focused on mitochondrial cytochrome *b*. There is also the advantage of using the same gene area for both PCR and LAMP, making the results obtained by both methods more comparable.

For both proposed methodologies, the successful amplification of pike DNA was verified. Amplification using qPCR with a probe takes approximately one hour. In the case of the LAMP method, the reaction takes 20–70 min depending on the lamplicon detection. When detection on agarose gel is used, the time required for electrophoresis must be included in addition. Similar results were published, for example Saull et al.^58^ who carried out the reaction at 63 °C for 60 min. They used a Mast Isoplex DNA amplification kit (Mast Group Ltd., UK) containing *Bst* polymerase for the detection of cod DNA. Also, Xiong et al.^55^ published comparable results with us using the LampMaster Mix Mastermix with *Bst* polymerase (Sangon, China); the reaction time was 60 min.

The use of LAMP, especially the real-time assay, thus provides a promising tool for very fast detection of pike DNA. According to the literature, there could be a further reduction in the time required for the LAMP reaction if loop primers are used. Nanayakkara and White,^62^ for example, have shown that the inclusion of these primers in the reaction accelerates the amplification by up to 10 min. Furthermore, another reaction mixture containing a polymerase with higher activity or providing better reaction conditions for the polymerase could be used. Ali et al.^56^ have achieved the detection of *Thunnus albacares* in less than 15 min when using the GspSSD Isothermal Mastermix (ISO-001) (OptiGene Ltd., UK). The same Mastermix was also used by Abdulmawjood et al.^63^ who obtained the results of the LAMP assay in 30 min. These results are in correspondence with the statements of the GspSSD Isothermal Mastermix manufacturer, OptiGene Ltd., which declares on its website (www.optigene.co.uk) that the amplification with their ISO-001 Mastermix is up to a third faster than the NEB WarmStart LAMP kit in the presence of 100 copies of human gDNA in the reaction.

Nevertheless, when comparing the estimated detection limits of the LAMP, real-time LAMP, and qPCR methods for pike, it was found that, for the designed primers and reaction conditions, the qPCR method is more sensitive. In the case of using the qPCR method, the detection limit was set at 0.1 ng of target DNA in the reaction. Moreover, it is possible to quantify up to 0.39 ng of pike DNA in the reaction mixture when the designed TaqMan probe is used. Compared to that, with the LAMP method, we can reliably detect 3.13 ng of template DNA in the reaction on the agarose gel and with SYBR Green detection based on color change of the mixture and 0.78 ng in real-time LAMP assay. In addition, it has been verified that it is possible to reliably detect 5 wt % of pike in mixed samples with qPCR method (Ct ≤ 30) and 10 wt % by LAMP (Figure S2). Content of pike in the sample less than 5 wt % was not tested. In the case of adulteration, we consider minor admixtures to be irrelevant, from an economic point of view.

Although LAMP is generally presented as a more sensitive method than PCR (e.g. ref.^64^), our results do not confirm this trend. Likewise to our results, Xiong et al.^53^ found in their study, where they proposed a LAMP and qPCR identification method for Atlantic salmon (*Salmo salar*), that the sensitivity of their qPCR assay was ten times higher than that of LAMP. In addition, it should be mentioned that, compared to qPCR detection, an indisputable advantage of the LAMP protocol proposed in our study is the possibility of detecting results “under sunlight”, which saves time and does not require expensive instruments.

Finally, both designed protocols were tested on fish products obtained from Czech markets. The samples tested included 3 specimens of pike, *E. lucius* (declared area of capture: Czech Republic), 2 skinless pike fillets (Russia), 4 dried gutted pike (Kazakhstan, Pacific Ocean), and pike caviar (not specified). The possibility of analysis of DNA from muscle, skin, and fin was verified; template DNA was amplified in all samples of commercial pike products. Only caviar DNA was amplified with a Ct value higher than 35 so it is on the edge of positivity in the qPCR assay, although lamplicons were visible on the agarose gel similar to other products in the LAMP method (Figure S3A).

Our results confirmed that both LAMP and PCR methods provide the ability to quickly detect the pike parvalbumin gene. Designed assays are very sensitive and can therefore serve as a quick warning for the presence of an allergen in food, as well as a tool for fish identification; the qPCR assay also enables accurate quantification of target DNA. The selected area of the parvalbumin gene allows the differentiation of *E. lucius*, *E. aquitanicus*, and *E. cisalpinus* only by amplicon sequencing. However, it should be possible to distinguish these European pike species from *E. niger* (which theoretically the F3 primer should not amplify and the Esox primers cover a sufficiently different area) and *E. masquinongy* (thanks to the Esox primers).

This study presents newly developed LAMP and qPCR assays for the efficient identification of *Esox* species and the detection of their parvalbumin gene. High specificity and sensitivity were confirmed for both assays. The advantage of the LAMP method is the fact that it does not require thermal cycling. This could allow the method to be used for in situ analysis outside the laboratory. Both developed methods have been successfully applied to the analysis of fresh and processed (dried, preserved in alcohol) fish tissue and pike caviar, which could be attractive for food and invasive species control programs. In addition, the necessary analysis of the pike parvalbumin gene, which encodes a major fish allergen, was successfully performed. Therefore, the work has contributed to increasing knowledge about the pike parvalbumin gene as well as to the practical outcome of protecting the food market and fish management. For future work, we recommend further investigation of the parvalbumin gene of individual pike species to design species-specific protocols for their differentiation.

## Supporting Information Available

Table. S1: the nucleotide sequences similarity of pike amplicons; Figure S1: electrophoreogram of PCR products obtained with *Esox*-spp primers; Figure S2: determination of detection limits for PCR and LAMP methods; Figure S3: demonstration of LAMP analysis of commercial pike products (PDF)
