# Supplementary material for: Parvalbumin Gene: A Valuable Marker for Pike Authentication and Allergen Risk Assessment
Source: J Agric Food Chem. 2024 May 23;72(22):12788–97. doi: 10.1021/acs.jafc.4c01410 (PMC11157528; doi:10.1021/acs.jafc.4c01410)
Supplement: Supplementary file 1 — jf4c01410_si_001.pdf [file jf4c01410_si_001.pdf]

Supplementary data to the article

Parvalbumin Gene: A Valuable Marker for Pike Authentication and Allergen Risk Assessment

Eliška Čermáková[a,b], Subham Mukherjee[a,c,d], Denisa Nováková[b], Kamila Zdeňková[b], Kateřina Demnerová[b]

[a]Department of Chemistry, Biochemistry and Food Microbiology, Food Research Institute Prague, Radiová 1285/7, 10231 Prague 10, Czech Republic.

[b]Department of Biochemistry and Microbiology, University of Chemistry and Technology, Prague, Technická 5, 166 28 Prague 6, Czech Republic.

[c]Institute for Environmental Studies, Faculty of Science, Charles University, Benatska 2, 128 01 Prague 2, Czech Republic.

[d]Lennard-Jones School of Chemical and Physical Sciences, Keele University, Staffordshire, ST5 5BG, United Kingdom

Supplementary files:

Tab. S1: The nucleotide sequences similarity of pike amplicons

Fig. S1: Electrophoreogram of PCR products obtained with *Esox*-spp primers

Fig. S2: Determination of detection limits for PCR and LAMP methods.

Fig. S3: Demonstration of LAMP analysis of commercial pike products.

Tab. S1: The nucleotide sequences similarity of pike amplicons (F3-B3 primers). LOC = LOC105005800 gene; ENSELU = ENSELUG00000036805.1 gene; Sanger = sequence was obtained by Sanger sequencing of the PCR product;* = part of reference genome obtained the from NCBI databases.

	*E. niger**	*E. masquiongy**	*E. lucius**	*E. lucius* (LOC)	*E. lucius* (ENSELU)	*E. lucius* (Sanger)	*E. aquitanicus* (Sanger)	*E. cisalpinus* (Sanger)
*E. niger**								
*E. masquiongy**	53.69%							
*E. lucius**	74.59%	63.83%						
E. lucius (LOC)	74.59%	63.83%	100.00%					
E. lucius (ENSELU)	74.59%	63.83%	100.00%	100.00%				
E. lucius (Sanger)	74.80%	64.04%	99.33%	99.33%	99.33%			
E. aquitanicus (Sanger)	75.00%	64.26%	98.65%	98.65%	98.65%	98.88%		
E. cisalpinus (Sanger)	75.41%	64.68%	99.10%	99.10%	99.10%	99.33%	99.55%	

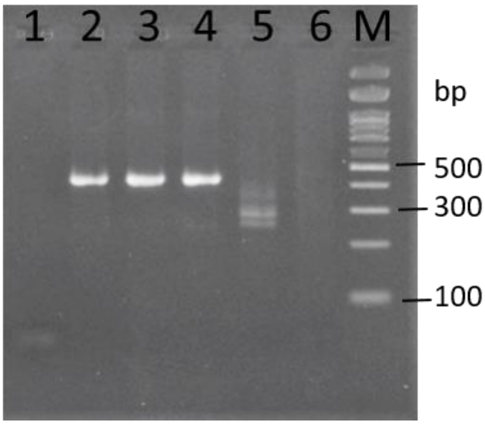

Fig.S1: Electrophoreogram of PCR products obtained with Esox-spp primers. Lines:1 = No template control, 2 = *Esox lucius*, 3 = *Esox aquitanicus*, 4 = *Esox cisalpinus*, 5 = *Cyprinus carpio*, 6 = *Salmo salar*, M = marker (100 bp ladder)

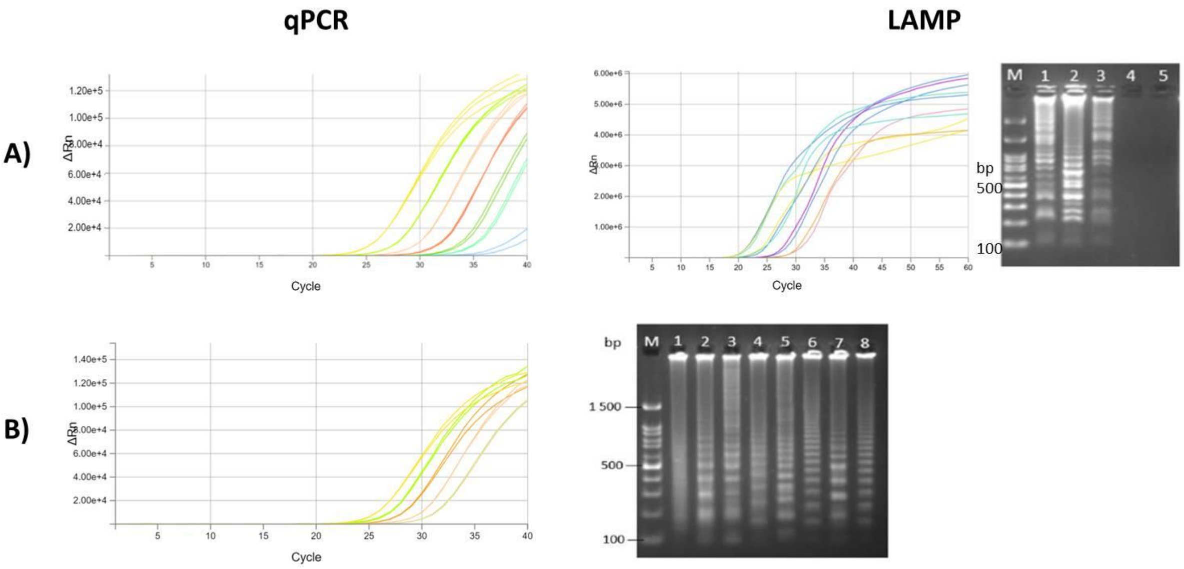

Fig.S2: Determination of detection limits for PCR (left) and LAMP (right) analysis of pike samples. A) Estimation of pike DNA detection limit based on a series of DNA dilution (4x) ranging from 100 – 0.02 ng for qPCR and 50 – 0.2 ng for both LAMP protocols; the line 5 in the electrophoresis is negative control, M = DNA Ladder CSL-MDNA 100bp. B) Determination of the detection limit of pike (*Esox lucius*, EL) admixture in cod (*Gadus morhua*, GM) muscle using qPCR (5, 20, 50, 70, and 100 wt. % of EL in the sample) and LAMP with electrophoretic detection, where the EL:GM tissue ratio (wt.%) in the mixture was in line: 1 = 5:95, 2 = 10:90, 3 = 20:80, 4 = 30:70, 5 = 40:60, 6 = 50:50, 7 = 60:40, 8 = 70:30; M = standard (DNA Ladder CSL-MDNA 100bp).

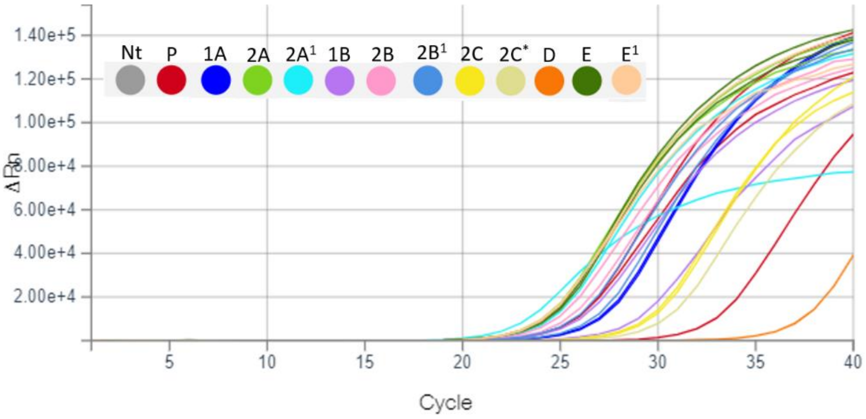

A)

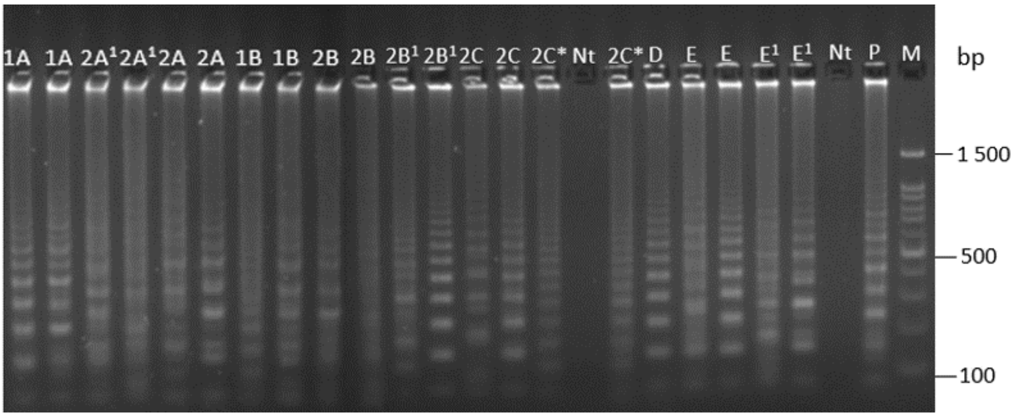

B)

Fig. S3: Demonstration of analysis of commercial pike products purchased in the market network of the Czech Republic. A) qPCR amplification curves, B) Electrophoreogram of lamplicons. Lines: M – standard (DNA Ladder CSL-MDNA 100bp), P = pike (*E. lucius*), Nt – no template control, 1 = filet of pike, 2 = dried pike, 2C = skin from dried pike (sample 2), 2C* = fin from dried pike (sample 2), D = pike caviar, E = dried pike; Letters A and B sign parallels, upper index 1 = muscle was dehydrated 15 min in distilled water before homogenization.
